# Effect of gestational age on clinical features in necrotizing enterocolitis-associated intestinal perforation

**DOI:** 10.3389/fped.2024.1452207

**Published:** 2025-01-06

**Authors:** Minming Chen, Wei Feng, Jinping Hou, Xiaohong Die, Zhenhua Guo, Yi Wang

**Affiliations:** Department of General & Neonatal Surgery, Children's Hospital of Chongqing Medical University, National Clinical Research Center for Child Health and Disorders, Ministry of Education Key Laboratory of Child Development and Disorders, Chongqing Key Laboratory of Structural Birth Defect and Reconstruction, Chongqing, China

**Keywords:** gestational age, clinical features, necrotizing enterocolitis, intestinal perforation, prognosis

## Abstract

**Purpose:**

To investigate the clinical features of necrotizing enterocolitis-associated intestinal perforation (NEC-IP) in neonates with different gestational ages (GAs). Furthermore, we also want to identify the risk factors of poor prognosis for these patients.

**Methods:**

The retrospective study of patients with NEC-IP was conducted with basic information, comorbidity, intraoperative findings, related treatment, and prognosis. According to the GA, patients were divided into three groups: early (GA: 28–<32 weeks, Group 1), mid-term (GA: 32–<34 weeks, Group 2), and late (GA: 34–<37 weeks, Group 3). The clinical features of the three groups were analyzed, and risk factors for poor prognosis were identified.

**Results:**

Of the 113 cases, the number of cases in Groups 1 to 3 was 36 (31.9%), 44 (38.9%), and 33 (29.2%), respectively; and the overall proportion of poor prognosis was 19.4% (22/113). For basic information, the birth weight of Group 1 was lower than that of Group 2 and Group 3, while the postnatal day at the time of surgery of NEC and the onset age were higher than that of Group 2 (onset age: G1 12.0[7.00;20.5], G2 9.00[4.00;13.0]; postnatal day at the time of surgery: G1 22.0[13.8;27.2], G2 13.0[8.00;21.0]) (*P* < 0.016). For comorbidity, the incidence of sepsis, coagulopathy, type of (congenital heart disease) CHD, and hypoproteinemia in Group 1 was higher than that in Group 2 (all *P* < 0.016), and the incidence of respiratory failure, hypoproteinemia in Group 1 was higher than that in Group 3 (all *P* < 0.016). For related treatment, the usage rate of vasoactive substances and mechanical ventilation in Group 1 was higher than that of Group 2 and Group 3 (all *P* < 0.016). By Lasso and Logistic regression analysis, we found that GA (OR: 0.274, 95%CI: 0.078–0.796), sepsis (OR: 7.955, 95%CI: 1.424–65.21), coagulopathy (OR: 19.51, 95%CI: 3.393–179.1), CHD (OR: 6.99, 95%CI: 1.418–54.83) and diseased bowel segment (OR: 2.804, 95%CI: 1.301–7.316) were the independent factors for poor prognosis (all *P* < 0.05).

**Conclusions:**

The clinical features of NEC-IP patients differ based on GA, particularly in terms of CHD type, postnatal day at the time of surgery, utilization of vasoactive substances, and prognosis. Furthermore, GA, sepsis, coagulopathy, CHD, and diseased bowel segment are independent factors for poor prognosis of patients with NEC-IP.

## 1 Introduction

Necrotizing enterocolitis (NEC) was one of the most common critical neonatal gastrointestinal emergencies with significant morbidity and mortality (1–3). Approximately 90% of affected patients were preterm infants (4). The study had found that the incidence of NEC in neonates with gestational age (GA) of less than 33 weeks was about 5.1% (1.3%–12.9%), and it increased with the decrease of GA (5). In addition, the mortality rate associated with NEC ranges from 15% to 30% (6), with a significantly higher risk in patients who developed necrotizing enterocolitis-associated intestinal perforation (NEC-IP) (1, 7).

Sharma et al. reported that there were significant differences in the incidence of NEC in early (GA: 28–<32weeks), middle (GA: 32–<34weeks), and late preterm patients (GA: 34–<37weeks) (8). GA was an influencing factor for the prognosis of NEC, that was, low GA was prone to poor prognosis (9). However, the effect of GA on clinical features in patients with NEC-IP and prognosis, remains unclear. Patients with NEC-IP are not uncommon clinically, and the mortality rate is high, especially among preterm patients, making it is of great clinical value to explore the related risk factors. Currently, there are many clinical studies investigating the prognostic factors of NEC, but there is a lack of research to judge the postoperative prognosis of NEC-IP. It is worth noting that the incidence of poor prognosis significantly increased when intestinal perforation (IP) was present in NEC (10). Therefore, more attention should be paid to these NEC-IP patients to improve the overall prognosis.

Therefore, this study aims to investigate the clinical features of NEC-IP in patients with different GAs and identify the risk factors for poor prognosis, providing the theoretical basis for improving the prognosis.

## 2 Methods

### 2.1 Study population

A retrospective review was performed on the clinical data of the patients with NEC-IP admitted to our department from January 2014 to March 2019. It was performed after the study protocol was approved by the Institutional Research Ethics Board of Children's Hospital affiliated Chongqing Medical University (Date: 09.28.2021/No: 329). The center operates a Level IV NICU, which provides advanced neonatal care, including specialized treatment for critically ill and premature infants. The inclusion criteria were: (1) patients with GA < 37 weeks; (2) patients diagnosed with NEC, with intraoperative proof of IP; (3) patients with complete clinical data and postoperative follow-up data. The exclusion criteria were as follows: (1) patients with congenital genetic metabolic-related diseases and chromosome diseases; (2) patients with congenital gastrointestinal malformations, such as Hirschsprung's disease, intestinal malrotation, and intestinal atresia; (3) these confirmed as “spontaneous isolated intestinal perforation” during the operation; (4) incomplete relevant variables. Patients were divided into three groups based on the range of GA: early (GA: 28−<32 weeks, Group 1), mid-term (GA: 32−<34 weeks, Group 2) and late (GA: 34−<37 weeks, Group 3).

### 2.2 Study design

Clinical data of the included patients were retrospectively collected in this study, including (1) basic information: gender, GA, birth weight, age of onset/surgery of NEC, and perinatal data; (2) preoperative comorbidities: hypoproteinemia, respiratory failure, sepsis, shock, liver/renal injury, coagulopathy, congenital heart disease (CHD), hyponatremia; (3) intraoperative findings: location of perforation (recorded as ≥2 segments if perforation site involves two or more of the colon/ileum/ileocecal at the same time); (4) treatment and prognosis. Poor prognosis was defined as NEC-related death during hospitalization or within the 3-month follow-up period after discharge (11).

### 2.3 Definition

NEC-IP Patients meeting the diagnosis of both NEC and IP were defined as NEC-IP. NEC was diagnosed according to (1) the pathognomonic findings on abdominal radiography, including pneumatosis intestinalis, portal venous gas, fixed dilated loops of bowel, and extraluminal air outside the bowel; and (2) the presence of one or more clinical findings, including intolerance to feeding, abdominal distension, abdominal tenderness, abdominal wall erythema/discoloration or abdominal mass, and bloody stools (12). IP was defined at least one of the following: (1) abdominal radiographs suggesting subphrenic free gas, encapsulated or circumscribed pneumoperitoneum; (2) abdominal ultrasonography indicating abdominal fluid accumulation, disappearance of peristalsis and intestinal necrosis; (3) abdominal puncture confirming the presence of gastrointestinal contents in the abdominal cavity; or (4) perforation found during surgery (4).

CHDs were divided into two groups based on the result of echocardiography: cyanotic CHD (right-to-left intracardiac or extracardiac shunts resulting in hypoxemia, erythrocytosis, and cyanosis), and non-cyanotic CHD (the rest malformations) (13, 14).

Hypoalbuminemia was defined as a serum albumin level below 25 g/L (15). Respiratory Failure in children was characterized by an inability to maintain normal gas exchange, reflected in arterial blood gas analysis (PaO2 < 60 mmHg or PaCO2 > 50 mmHg). Hyponatremia was defined as a serum sodium concentration of less than 135 mmol/L (16). Shock was primarily diagnosed based on clinical manifestations, including hypotension, tachycardia, and compromised peripheral circulation, such as cold extremities and reduced urine output. Liver and Kidney Injury in children were identified by abnormal liver enzymes (for liver injury) or elevated serum creatinine (for kidney injury). Coagulopathy was considerate when one of the disturbs were present: platelet count (PLT) less than 100 (× 10^9^ /L), APTT superior to 45.4 s, and PT-INR superior to 1.3. Sepsis was diagnosed when a pathogen was isolated from either blood or cerebrospinal fluid, and infants exhibiting infectious manifestations were treated with antibiotics for at least five days (17).

### 2.4 Statistical analysis

Excel 2007 software was used to double-check and enter the recorded data, and Statistical Package for Social Sciences 25.0 software was used for statistical analysis. Continuous data were assessed for normality using the Shapiro–Wilk test. Data following a normal distribution were expressed as the mean ± standard deviation (SD) and analyzed using analysis of variance (ANOVA) for multi-group comparisons. Data that did not follow a normal distribution were presented as the median and interquartile range (IQR) and analyzed using the Kruskal–Wallis test for multi-group comparisons. Categorical data were expressed as *n* (%), and Fisher's exact test or chi-squared test, as appropriate, was used for comparison. Statistical tests were corrected for multiple comparisons using Bonferroni correction with a corrected *P*-value of 0.016. Covariate screening was performed using LASSO analysis with the “glmnet” package in R 4.1.3 software, followed by multivariate Logistic regression analysis to identify independent factors associated with poor prognosis in NEC-IP patients. *P* value < 0.05 was regarded as significant.

## 3 Results

### 3.1 General data

In this study, 129 patients were diagnosed with NEC-IP. Among these cases, 8 had a GA of less than 28 weeks, 5 were associated with intestinal malrotation, and 3 presented with intestinal atresia. Only three cases of IP in full-term NEC patients were identified during retrospective data collection. Due to the small sample size of full-term patients, only preterm NEC patients were analyzed. Ultimately, a total of 113 patients met the inclusion criteria and were enrolled in the study. Out of 113 cases, the average GA and birth weight were 32.29 ± 2.09 weeks, and 1,965.5 ± 542.5 grams, respectively. The incidence was essentially similar in males and females, and the male-to-female ratio was 0.98 (56:57 cases). Additionally, the median onset age was 11 days, and the media postnatal day at the time of surgery was 15 days. During surgery, we found that the most commonly affected segment in IP was the colon (40.7%), followed by the ileum (37.2%), involvement of ≥2 segments (16.8%), and the ileocecal region (5.3%). It should be noted that the overall proportion of poor prognosis was 19.4% (22/113) (Table 1). The all-cause mortality in this study was 30.1% (34/113) and the NEC-related mortality was 19.4 (22/113).

**Table 1:** Comparison of general characteristics among NEC-IP patients at different gestational ages.

	[All] *N* = 113	G1 *N* = 36	G2 *N* = 44	G3 *N* = 33	*P* value
Gender (*n*/%)					0.290
Male	57 (50.4%)	15 (41.7%)	22 (50.0%)	20 (60.6%)	
Female	56 (49.6%)	21 (58.3%)	22 (50.0%)	13 (39.4%)	
Low birth weight (grams, *n*/%)					**0** **.** **004**
<1,000	3 (2.65%)	2 (5.56%)	0 (0.00%)	1 (3.0%)	
1,000–<1,500	31 (27.4%)	17 (47.2%)	9 (20.5%)	5 (15.2%)	
1,500–<2,500	79 (69.9%)	17 (47.2%)	34 (77.2%)^b^	24 (72.7%)^b^	
≥2,500	4 (3.5%)	0 (0.0%)	1 (2.3%)	3 (9.1%)	
Pregnancy (*n*/%)					0.366
Single	73 (64.6%)	20 (55.6%)	31 (70.5%)	22 (66.7%)	
Multiple	40 (35.4%)	16 (44.4%)	13 (29.5%)	11 (33.3%)	
Vaginal delivery (*n*/%)					**0**.**032**
Yes	34 (30.1%)	14 (38.9%)	7 (15.9%)	13 (39.4%)	
No	79 (69.9%)	22 (61.1%)	37 (84.1%)	20 (60.6%)	
PROM (*n*/%)					0.479
No	67 (59.3%)	19 (52.8%)	29 (65.9%)	19 (57.6%)	
Yes	46 (40.7%)	17 (47.2%)	15 (34.1%)	14 (42.4%)	
MSAF (*n*/%)					0.058
No	100 (88.5%)	33 (91.7%)	35 (79.5%)	32 (97.0%)	
Yes	13 (11.5%)	3 (8.33%)	9 (20.5%)	1 (3.03%)	
FIUD (*n*/%)					0.243
No	93 (82.3%)	32 (88.9%)	33 (75.0%)	28 (84.8%)	
Yes	20 (17.7%)	4 (11.1%)	11 (25.0%)	5 (15.2%)	
PIH (*n*/%)					0.739
No	84 (74.3%)	28 (77.8%)	31 (70.5%)	25 (75.8%)	
Yes	29 (25.7%)	8 (22.2%)	13 (29.5%)	8 (24.2%)	
GDM (*n*/%)					0.670
No	82 (72.6%)	25 (69.4%)	34 (77.3%)	23 (69.7%)	
Yes	31 (27.4%)	11 (30.6%)	10 (22.7%)	10 (30.3%)	
ICP (*n*/%)					0.523
No	87 (77.0%)	30 (83.3%)	32 (72.7%)	25 (75.8%)	
Yes	26 (23.0%)	6 (16.7%)	12 (27.3%)	8 (24.2%)	
Maternal age (*n*/%)					0.310
<35	77 (68.1%)	21 (58.3%)	32 (72.7%)	24 (72.7%)	
≥35	36 (31.9%)	15 (41.7%)	12 (27.3%)	9 (27.3%)	
EN (*n*/%)					0.383
No	17 (15.0%)	8 (22.2%)	5 (11.4%)	4 (12.1%)	
Yes	96 (85.0%)	28 (77.8%)	39 (88.6%)	29 (87.9%)	
Onset age^a^^(day)^	11.0 [6.00;14.0]	12.0 [7.00;20.5]	9.00 [4.00;13.0]^b^	10.0 [7.00;13.0]	0.055
Postnatal day at the time of surgery^a^^(day)^	15.0 [9.00;23.0]	22.0 [13.8;27.2]	13.0 [8.00;21.0]^b^	13.0 [9.00;18.0]	**0**.**004**
Prognosis (*n*/%)					**<0**.**001**
Good	91 (80.5%)	19 (52.8%)	42 (95.5%)	30 (90.9%)	
Poor	22 (19.5%)	17 (47.2%)	2 (4.55%)^b^	3 (9.09%)^b^	

PROM, premature rupture of membrane; MSAF, meconium-stained amniotic fluid; FIUD, fetal intrauterine distress; PIH, pregnancy induced hypertension; GDM, gestational diabetes mellitus; ICP, intrahepatic cholestasis of pregnancy; EN, enteral nutrition.

Bold indicates a *p*-values <0.05.

^a^ Values are presented as median [IQR, interquartile range].

^b^ Indicates a statistical difference with the Group 1, and.

^c^ Indicates a statistical difference with the Group 2.

### 3.2 Clinical features of patients with different GAs

The onset age and postnatal day at the time of surgery in Group 1 were significantly higher than those in Group 2 (median onset age: 12 days vs. 9 days; median postnatal day at surgery: 22 days vs. 13 days, both *P* < 0.016). However, no statistically significant differences were observed in onset age and postnatal day at surgery between Group 3 and Groups 1/2 (all *P* > 0.016). As was expected the birth weight in Group 1 was lower than that in Group2 and Group3 (*P* < 0.016). Furthermore, the incidence of poor prognosis in Group 1 (17 cases, 47.2%) was significantly higher than that in Group 2 (2 cases, 4.55%) and Group 3 (3 cases, 9.09%) (both *P* < 0.016).

Regarding comorbidities, NEC-IP was most commonly accompanied by CHD (91.2%), followed by hypoproteinemia (50.4%), sepsis (44.2%), coagulopathy (40.7%), and respiratory failure (42.5%) (Table 2). The incidence of respiratory failure, sepsis, renal injury, coagulopathy, and hypoproteinemia were different among different GAs (*P* < 0.05). Compared with Group 1, the incidence of sepsis (61.1% vs. 31.8%), coagulopathy (50.0% vs. 34.1%), cyanotic CHD (47.2% vs. 11.4%), hypoproteinemia (72.2% vs. 38.6%) was higher than that in Group2, while the incidence of respiratory failure (55.6% vs. 21.2%), hypoproteinemia (72.2% vs. 42.4%) was higher than that in Group3 (all *P* < 0.016).

**Table 2:** Comparison of the complications among NEC-IP patients at different gestational ages.

	[All] *N* = 113	G1 *N* = 36	G2 *N* = 44	G3 *N* = 33	*P* value
Sepsis (*n*/%)					**0** **.** **031**
NO	63 (55.8%)	14 (38.9%)	30 (68.2%)	19 (57.6%)	
YES	50 (44.2%)	22 (61.1%)	14 (31.8%)^a^	14 (42.4%)	
Respiratory failure (*n*/%)					**0**.**010**
No	65 (57.5%)	16 (44.4%)	23 (52.3%)	26 (78.8%)	
Yes	48 (42.5%)	20 (55.6%)	21 (47.7%)	7 (21.2%)^a^	
Coagulopathy (*n*/%)					0.348
No	67 (59.3%)	18 (50.0%)	29 (65.9%)	20 (60.6%)	
Yes	46 (40.7%)	18 (50.0%)	15 (34.1%)	13 (39.4%)	
Renal injury (*n*/%)					0.275
No	77 (68.1%)	21 (58.3%)	31 (70.5%)	25 (75.8%)	
Yes	36 (31.9%)	15 (41.7%)	13 (29.5%)	8 (24.2%)	
Liver injury (*n*/%)					0.315
No	87 (77.0%)	25 (69.4%)	34 (77.3%)	28 (84.8%)	
Yes	26 (23.0%)	11 (30.6%)	10 (22.7%)	5 (15.2%)	
Hypoproteinemia (*n*/%)					**0**.**006**
No	56 (49.6%)	10 (27.8%)	27 (61.4%)	19 (57.6%)	
Yes	57 (50.4%)	26 (72.2%)	17 (38.6%)^a^	14 (42.4%)^a^	
Hyponatremia (*n*/%)					0.281
No	78 (69.0%)	22 (61.1%)	34 (77.3%)	22 (66.7%)	
Yes	35 (31.0%)	14 (38.9%)	10 (22.7%)	11 (33.3%)	
CHD (*n*/%)					**0**.**002**
No	10 (8.8%)	4 (11.1%)	3 (6.82%)	3 (9.09%)	
Non-cyanotic type	75 (66.4%)	15 (41.7%)	36 (81.8%)^a^	24 (72.7%)	
Cyanotic type	28 (24.8%)	17 (47.2%)	5 (11.4%)	6 (18.2%)	
Diseased bowel segment (*n*/%)					0.431
Ileum	42 (37.2%)	9 (25.0%)	19 (43.2%)	14 (42.4%)	
Colon	46 (40.7%)	15 (41.7%)	18 (40.9%)	13 (39.4%)	
Ileocecal region	6 (5.3%)	2 (5.5%)	2 (4.5%)	2 (6.1%)	
≥2 segments	19 (16.8%)	10 (27.8%)	5 (11.4%)	4 (12.1%)	

CHD, congenital heart disease.

Bold indicates a *p*-values <0.05.

^a^ Indicates a statistical difference with Group 1.

^b^ Indicates a statistical difference with Group 2.

According to the intraoperative findings, we found that there was no significant difference in the diseased bowel segment among patients with different GAs. For related treatment, there were significant differences in the utilization rate of vasoactive substances, mechanical ventilation, and blood transfusion among different GAs (*P* < 0.05) (Table 3). Furthermore, the utilization rate of vasoactive substances (61.1% vs. 34.1% vs. 27.3%; *P* < 0.016) and mechanical ventilation (75.0% vs. 43.2% vs. 21.2%; *P* < 0.016) in Group1 was higher than that in Group2 and Group3, respectively.

**Table 3:** Comparison of the treatment among NEC-IP patients at different gestational ages.

	[All] *N* = 113	G1 *N* = 36	G2 *N* = 44	G3 *N* = 33	*P* value
Probiotics (*n*/%)					0.160
No	42 (37.2%)	10 (27.8%)	21 (47.7%)	11 (33.3%)	
Yes	71 (62.8%)	26 (72.2%)	23 (52.3%)	22 (66.7%)	
Albumin infusion (n/%)					0.085
No	57 (50.4%)	16 (44.4%)	19 (43.2%)	22 (66.7%)	
Yes	56 (49.6%)	20 (55.6%)	25 (56.8%)	11 (33.3%)	
Plasma transfusion (*n*/%)					0.176
No	87 (77.0%)	24 (66.7%)	37 (84.1%)	26 (78.8%)	
Yes	26 (23.0%)	12 (33.3%)	7 (15.9%)	7 (21.2%)	
Gamma globulin infusion (*n*/%)					0.523
No	94 (83.2%)	28 (77.8%)	37 (84.1%)	29 (87.9%)	
Yes	19 (16.8%)	8 (22.2%)	7 (15.9%)	4 (12.1%)	
Blood transfusion (*n*/%)					**0** **.** **041**
No	66 (58.4%)	15 (41.7%)	28 (63.6%)	23 (69.7%)	
Yes	47 (41.6%)	21 (58.3%)	16 (36.4%)	10 (30.3%)	
Mechanical ventilation (*n*/%)					**<0**.**001**
No	60 (53.1%)	9 (25.0%)	25 (56.8%)	26 (78.8%)	
Yes	53 (46.9%)	27 (75.0%)	19 (43.2%)^b^	7 (21.2%)^b^	
Vasoactive substances (*n*/%)					**0**.**009**
No	67 (59.3%)	14 (38.9%)	29 (65.9%)	24 (72.7%)	
Yes	46 (40.7%)	22 (61.1%)	15 (34.1%)^b^	9 (27.3%)^b^	

Bold indicates a *p*-values <0.05.

^a^ Values are presented as median [IQR, interquartile range].

^b^ Indicates a statistical difference with the Group 1.

^c^ Indicates a statistical difference with the Group 2.

### 3.3 Risk factors for poor prognosis

**Figure 1:**
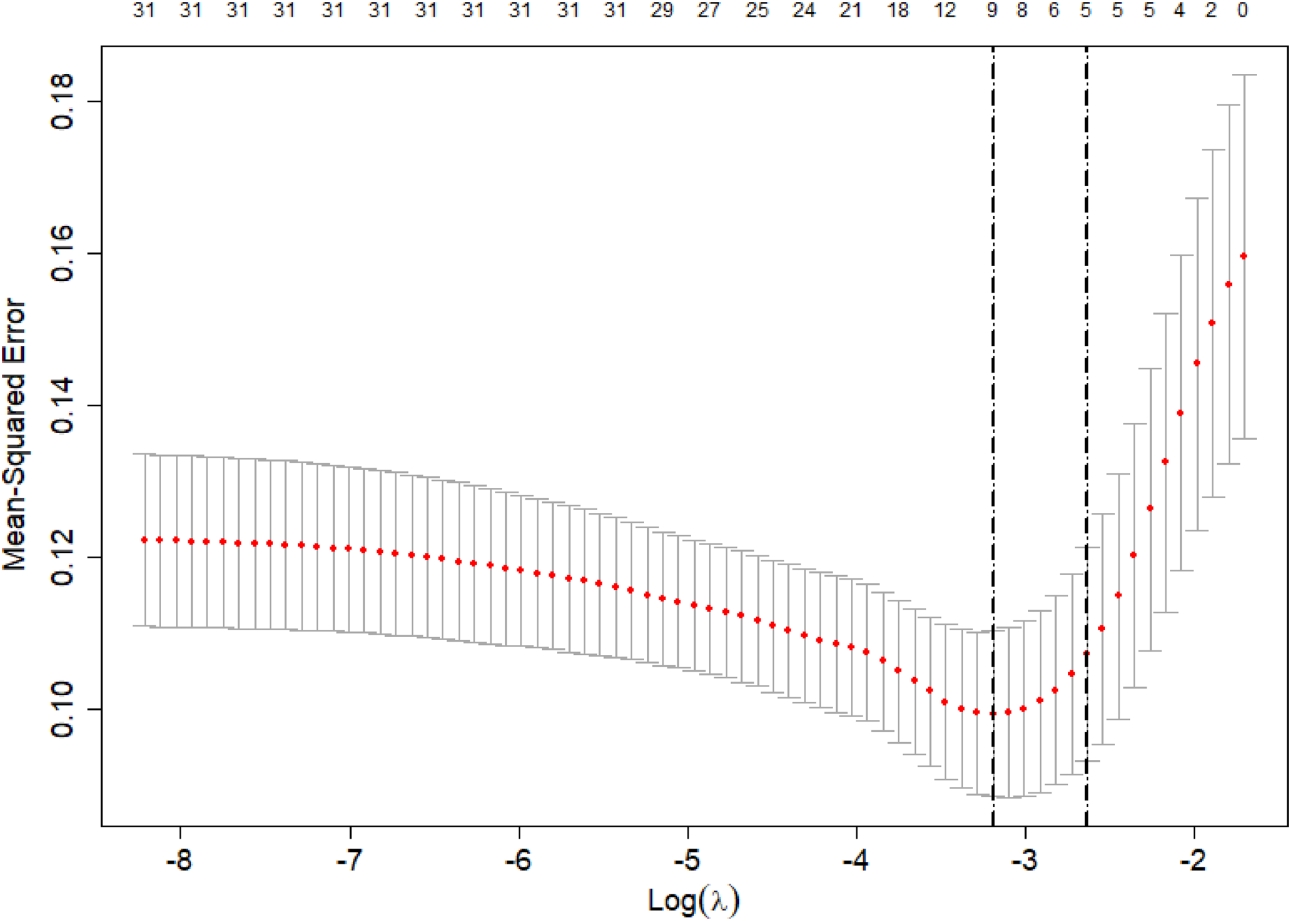
LASSO regression cross-validation results.

**Figure 2:**
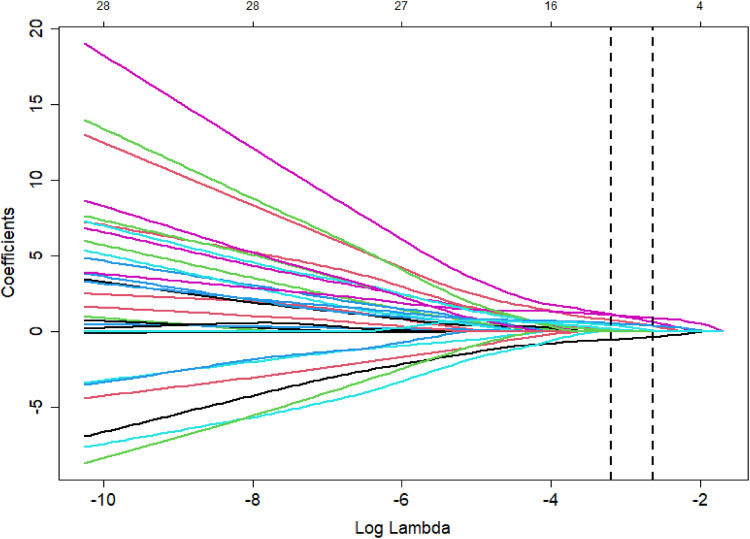
Coefficient path of LASSO regression.

The poor prognosis of NEC-IP patients (assignment: no = 0, yes = 1) was used as the dependent variable, and the influencing factors were screened by LASSO regression (Tables 4–6). The screened influence factors included GA, sepsis, coagulopathy, CHD, diseased bowel segment, and blood transfusion (Figures 1, 2). These factors were used as independent variables for multivariate Logistic regression analysis. The results showed that GA (OR: 0.274, 95%CI: 0.078–0.796O), sepsis (OR: 7.955, 95%CI: 1.424–65.21), coagulopathy (OR: 19.51, 95%CI: 3.393–179.1), CHD (OR: 6.99, 95%CI: 1.418–54.83) and diseased bowel segment (OR: 2.804, 95%CI: 1.301–7.316) were independent factors for poor prognosis (Table 7). The Nagelkerke R-squared and Akaike Information Criterion (AIC) values for the model were 0.726 and 48.789, respectively.

**Table 4:** Impact of perinatal factors on patient prognosis.

	[All] *N* = 113	Good prognosis (*N* = 91, 80.6%)	Poor prognosis (*N* = 22, 19.4%)	*P* value
Gender (*n*/%)				0.777
Male	57 (50.4%)	47 (51.6%)	10 (45.5%)	
Female	56 (49.6%)	44 (48.4%)	12 (54.5%)	
Gestational age (weeks, *n*/%)				**<0** **.** **001**
<32	36 (31.9%)	19 (20.9%)	17 (77.3%)	
32–<34	44 (38.9%)	42 (46.2%)	2 (9.09%)	
34–<37	33 (29.2%)	30 (33.0%)	3 (13.6%)	
Pregnancy (*n*/%)				0.723
Single	73 (64.6%)	60 (65.9%)	13 (59.1%)	
Multiple	40 (35.4%)	31 (34.1%)	9 (40.9%)	
Low birth weight (grams, *n*/%)				0.083
<1,000	3 (2.65%)	1 (1.10%)	2 (9.09%)	
1,000–<1,500	31 (27.4%)	27 (29.7%)	4 (18.2%)	
1,500–<2,500	79 (69.9%)	59 (64.8%)	16 (72.7%)	
≥2,500	4 (3.5%)	4 (4.4%)	0 (0.00%)	
Vaginal delivery (*n*/%)				0.562
Yes	34 (30.1%)	29 (31.9%)	5 (22.7%)	
No	79 (69.9%)	62 (68.1%)	17 (77.3%)	
PROM (*n*/%)				0.826
No	67 (59.3%)	53 (58.2%)	14 (63.6%)	
Yes	46 (40.7%)	38 (41.8%)	8 (36.4%)	
MSAF (*n*/%)				0.457
No	100 (88.5%)	79 (86.8%)	21 (95.5%)	
Yes	13 (11.5%)	12 (13.2%)	1 (4.55%)	
FIUD (*n*/%)				0.354
No	93 (82.3%)	73 (80.2%)	20 (90.9%)	
Yes	20 (17.7%)	18 (19.8%)	2 (9.09%)	
PIH (*n*/%)				1.000
No	84 (74.3%)	68 (74.7%)	16 (72.7%)	
Yes	29 (25.7%)	23 (25.3%)	6 (27.3%)	
GDM (*n*/%)				0.435
No	82 (72.6%)	68 (74.7%)	14 (63.6%)	
Yes	31 (27.4%)	23 (25.3%)	8 (36.4%)	
ICP (*n*/%)				1.000
No	87 (77.0%)	70 (76.9%)	17 (77.3%)	
Yes	26 (23.0%)	21 (23.1%)	5 (22.7%)	
Maternal age (*n*/%)				0.447
<35	7 (68.1%)	64 (70.3%)	13 (59.1%)	
≥35	36 (31.9%)	27 (29.7%)	9 (40.9%)	
EN (*n*/%)				1.000
No	17 (15.0%)	14 (15.4%)	3 (13.6%)	
Yes	96 (85.0%)	77 (84.6%)	19 (86.4%)	
Onset age^a^^(day)^	11.0 [6.00; 14.0]	10.0 [6.50; 14.0]	11.5 [6.25; 16.8]	0.613
Postnatal day at the time of surgery^a^^(day)^	15.0 [9.00; 23.0]	15.0 [9.00; 23.0]	15.5 [8.50; 22.8]	0.802

PROM, premature rupture of membrane; MSAF, meconium-stained amniotic fluid; FIUD, fetal intrauterine distress; PIH, pregnancy induced hypertension; GDM, gestational diabetes mellitus; ICP, intrahepatic cholestasis of pregnancy; EN, enteral nutrition.

Bold indicates a *p*-values <0.05.

^a^ Values are presented as median [IQR, interquartile range].

**Table 5:** Impact of complications on patient prognosis.

	[All] *N* = 113	Good prognosis (*N* = 91, 80.6%)	Poor prognosis (*N* = 22, 19.4%)	*P* value
Sepsis (*n*/%)				**0** **.** **001**
No	63 (55.8%)	58 (63.7%)	5 (22.7%)	
Yes	50 (44.2%)	33 (36.3%)	17 (77.3%)	
Respiratory failure (*n*/%)				0.941
No	65 (57.5%)	53 (58.2%)	12 (54.5%)	
Yes	48 (42.5%)	38 (41.8%)	10 (45.5%)	
Coagulopathy (*n*/%)				**<0**.**001**
No	67 (59.3%)	62 (68.1%)	5 (22.7%)	
Yes	46 (40.7%)	29 (31.9%)	17 (77.3%)	
Renal injury (*n*/%)				0.447
No	77 (68.1%)	64 (70.3%)	13 (59.1%)	
Yes	36 (31.9%)	27 (29.7%)	9 (40.9%)	
Liver injury (*n*/%)				0.169
No	87 (77.0%)	73 (80.2%)	14 (63.6%)	
Yes	26 (23.0%)	18 (19.8%)	8 (36.4%)	
Hypoproteinemia (*n*/%)				**0**.**036**
No	56 (49.6%)	50 (54.9%)	6 (27.3%)	
Yes	57 (50.4%)	41 (45.1%)	16 (72.7%)	
Hyponatremia (*n*/%)				**0**.**016**
No	78 (69.0%)	68 (74.7%)	10 (45.5%)	
Yes	35 (31.0%)	23 (25.3%)	12 (54.5%)	
CHD (*n*/%)				**<0**.**001**
No	10 (8.85%)	10 (11.0%)	0 (0.00%)	
Non-cyanotic	75 (66.4%)	68 (74.7%)	7 (31.8%)	
Cyanotic	28 (24.8%)	13 (14.3%)	15 (68.2%)	
Diseased bowel segment (*n*/%)				**<0**.**001**
Ileum	42 (37.2%)	39 (42.9%)	3 (13.6%)	
Colon	46 (40.7%)	38 (41.8%)	8 (36.4%)	
Ileocecal region	6 (5.31%)	6 (6.59%)	0 (0.00%)	
≥2 segments	19 (16.8%)	8 (8.79%)	11 (50.0%)	

CHD, congenital heart disease.

Bold indicates a *p*-values <0.05.

**Table 6:** Impact of treatment on patient prognosis.

	[All] *N* = 113	Good prognosis (*N* = 91, 80.6%)	Poor prognosis (*N* = 22, 19.4%)	*P* value
Probiotics (*n*/%)				0.874
No	42 (37.2%)	33 (36.3%)	9 (40.9%)	
Yes	71 (62.8%)	58 (63.7%)	13 (59.1%)	
Albumin infusion (*n*/%)				0.777
No	57 (50.4%)	47 (51.6%)	10 (45.5%)	
Yes	56 (49.6%)	44 (48.4%)	12 (54.5%)	
Plasma transfusion (*n*/%)				0.052
No	87 (77.0%)	74 (81.3%)	13 (59.1%)	
Yes	26 (23.0%)	17 (18.7%)	9 (40.9%)	
Gamma globulin infusion (*n*/%)				0.200
No	94 (83.2%)	78 (85.7%)	16 (72.7%)	
Yes	19 (16.8%)	13 (14.3%)	6 (27.3%)	
Blood transfusion (*n*/%)				**0** **.** **010**
No	66 (58.4%)	59 (64.8%)	7 (31.8%)	
Yes	47 (41.6%)	32 (35.2%)	15 (68.2%)	
Mechanical ventilation (*n*/%)				0.130
No	60 (53.1%)	52 (57.1%)	8 (36.4%)	
Yes	53 (46.9%)	39 (42.9%)	14 (63.6%)	
Vasoactive substances (*n*/%)				**0**.**028**
No	67 (59.3%)	59 (64.8%)	8 (36.4%)	
Yes	46 (40.7%)	32 (35.2%)	14 (63.6%)	

Bold indicates a *p*-values <0.05.

^a^ Values are presented as median [IQR, interquartile range].

**Table 7:** Logistic regression screening of independent influencing factors of poor prognosis in NEC-IP patients.

Characteristics	β	SE	OR	95%CI	*P*
Gestational age	−1.294	0.57,657	0.274	0.274 (0.078–0.796)	**0**.**025**
Sepsis	2.074	0.9483	7.955	7.955 (1.424–65.21)	**0**.**029**
Coagulopathy	2.971	0.9855	19.516	19.51 (3.393–179.1)	**0**.**003**
CHD^a^	1.945	0.91182	6.99	6.99 (1.418–54.83)	**0**.**033**
Diseased bowel segment^b^	1.031	0.42939	2.804	2.804 (1.301–7.316)	**0**.**016**
Blood transfusion	1.717	0.97661	5.57	5.57 (0.923–47.77)	0.079

β, regression coefficient; SE, standard error; OR, odds ratio; 95%CI, 95% confidence interval; CHD, congenital heart disease.

Bold indicates a *p*-values <0.05.

^a^ NO CHD as the subvariable.

^b^ Ileum perforation as the subvariable.

## 4 Discussion

NEC was typically caused by intestinal inflammation. In the absence of early and effective intervention, the inflammation progressively worsened, extending through the mucosal, submucosal, muscular, and serosal layers of the intestine, ultimately leading to IP (18–20). IP was a life-threatening complication of NEC patients (4), with a mortality rate of nearly 76% (21). However, there were few studies on the poor prognosis of NEC-IP. Although necrotizing enterocolitis with intestinal perforation (NEC-IP) and spontaneous intestinal perforation (SIP) may present with similar clinical features, studies, including the The Necrotizing Enterocolitis Surgery Trial (NEST), identified significant distinctions between the two conditions. These findings suggested that NEC-IP and SIP differ fundamentally in both their pathogenic mechanisms and clinical manifestations (22, 23). This study included only pediatric patients with NEC-IP, all of whom underwent surgical treatment. During surgery, the intestinal pathology was assessed to confirm NEC-IP rather than SIP. GA was known to be a risk factor for NEC progressing to IP (24). The immune function, intestinal maturity, and intestinal colonizing microbiota were different for patients of different GAs (4, 25, 26).

Comparing early preterm patients with middle and late preterm patients, we found that early preterm patients had lower birth weight, later onset age, and later postnatal day at the time of surgery, and were more likely to have ≥2 segments of IPs. In early preterm patients, there was a higher incidence of respiratory failure, sepsis, CHD, and hypoproteinemia. Additionally, the utilization rate of vasoactive substances, mechanical ventilation, and blood transfusion was also higher in this group. However, this difference was not evident in the comparison of middle and late preterm patients. Although kidney and liver failure showed no significant differences between the intermediate and late-stage groups, the incidence of lung failure was notably higher in the intermediate group (47%) compared to the late-stage group (20%). This finding may suggest a higher risk of respiratory system injury, potentially due to bronchopulmonary dysplasia, even among moderately preterm infants.

In this study, the incidence of poor prognosis was 19.4%. Studies have suggested that the risk factors for NEC patients include low birth weight, small GA, mechanical ventilation, premature rupture of membranes, sepsis, shock, pulmonary surfactant drugs, and cesarean section (9, 27–29). By Lasso and Logistic regression analysis, we found that GA, sepsis, coagulopathy, CHD, and diseased bowel segment were the independent factors for poor prognosis in patients with NEC-IP.

As shown in our results, 61.1% of early preterm patients with NEC-IP had sepsis. It was reported that early preterm patients were more susceptible to sepsis than full-term patients (28). Generally, NEC-IP patients exhibited an underdeveloped immune system, an unstable intestinal microbiota, and an increased susceptibility to intestinal barrier damage (27, 30). With sepsis, bacteria multiplied in the blood and produced large amounts of toxins. These toxins acted on immature and damaged intestinal epithelial cells, stimulating the production of TNF-α, IL-8, PAF, and other cytokines, causing a cytokine-mediated inflammatory cascade (6). This further aggravates intestinal damage, resulting in a significant increase in the risk of intestinal necrosis, IP, peritonitis, and even septic shock, which could endanger the patient's life (31, 32). In this study, the incidence of septic shock in patients with NEC-IP was 5.8%. For patients with septic shock, hypoxia further aggravated the degree of intestinal mucosal ischemia and intestinal microcirculation disturbance (33). This led to the deterioration of the patient's intestinal condition and aggravation of the condition, which in turn will affect the prognosis.

Furthermore, coagulopathy was an independent risk factor for patients with NEC-IP. Patients with NEC-IP often exhibited abnormal gene expression of coagulation and anticoagulation proteins, which enhanced coagulation function and damaged the fibrinolytic system, leaving NEC-IP patients in a net pro-coagulation state (34). Moreover, relevant studies had confirmed that coagulopathy and mesenteric thrombosis were common in patients with NEC (35). Both of them aggravated intestinal tissue ischemia and intestinal mucosal epithelial necrosis, increasing the permeability of the intestinal wall, which led to extensive intestinal necrosis and multiple organ dysfunction syndrome (MODS), etc (35). Hutter et al. (36) and Sonntag et al. (37) reported disseminated intravascular coagulation (DIC) in 35% and 28% of patients with NEC, respectively. In our study, we found that 40.7% of patients with NEC-IP had abnormal coagulation, consistent with previous findings. Therefore, it was crucial to regularly assess coagulation function in NEC-IP patients and implement early interventions to correct coagulopathy, aiming to reduce the risk of poor prognosis.

This study found that GA was a risk factor for poor prognosis in patients with NEC-IP. The mortality rate of early preterm patients was 47.2% in this study, which was significantly higher than that of middle and late preterm patients. Small GA patients had a high probability of NEC, neonatal asphyxia, brain injury, and respiratory distress syndrome (38, 39). We found that the smaller the GA patients were, the higher the incidence of respiratory failure, sepsis, CHD, and hypoproteinemia, and the more frequently blood transfusion, ventilator support, and other medical methods were used. The reason may have been that their intestinal tracts were immature and their intestinal flora was not stabilized (2, 6, 40). In addition, the results indicated that the diseased bowel segment was also an influencing factor for the poor prognosis of NEC-IP. The reason may have been that patients with multiple-segment perforation had more severe conditions, more intestinal segments were removed than those with single-segment perforation, and the incidence of short-term and long-term complications was higher.

In this retrospective study, 24.8% of NEC-IP patients had cyanotic congenital heart disease (CHD), with a poor prognosis rate of 68.2%. Patent ductus arteriosus (PDA) accounted for 20.4% (21/103) of the CHD cases. The incidence of poor prognosis was much higher than the NEC patients combined with non-cyanotic type CHD (31.8%) and no CHD (0.00%). Multiple studies had established the association between CHD and an increased risk of NEC (38, 41–44). Specifically, research had shown that the presence of a PDA in NEC patients tripled the risk of severe NEC and increased the risk of mortality by fivefold (45). Patients with NEC-IP combined with cyanotic CHD had a significantly increased risk of poor prognosis, as the study reported, this may be related to CHD whose hemodynamic disorder can lead to intestinal damage. Recently, studies had been conducted on its pathogenesis. The main mechanism was a combination of mesenteric hypoperfusion and hypoxia-induced inflammation (42, 46). The colon and distal ileum were most commonly involved in these patients, which was related to the tendency of hypoperfusion in this area (27, 42, 47). And for these patients, their heart disease was not treated, which meant that after surgery, the blood supply of the intestine would still be affected, leading to intestinal inflammation and nutrient absorption disorders. Hence, it was crucial to ensure effective communication with the guardians of NEC-IP patients with CHD regarding the condition and associated risks before surgery. Furthermore, allocating limited medical resources to those at the highest risk should be prioritized.

## 5 Limitation

However, there are some limitations to this study. Firstly, considering that our hospital is the largest national clinical research center for child health and disorders in southwest China, the patients were in a relatively serious condition. Secondly, inherent biases were inevitable given the single-center retrospective study with a relatively small sample size. Therefore, further verification is needed for a multi-center, large sample, and multidisciplinary prospective cohort study.

## 6 Conclusions

We found most NEC-related IP in patients born at GA 32–34. The clinical features of patients with different GAs were different, especially in terms of the type of CHD, onset time of IP, utilization of vasoactive substances, and prognosis. Furthermore, GA, sepsis, coagulopathy, CHD, and diseased bowel segment were independent factors for the prognosis of the patients with NEC-IP. Therefore, recognizing the influencing factors for poor prognosis of NEC-IP facilitates our early identification of those at risk and leads to individualized interventions to improve the clinical outcome.

## Data Availability

The original contributions presented in the study are included in the article/Supplementary Material, further inquiries can be directed to the corresponding author.
